# Two Antimycin A Analogues from Marine-Derived Actinomycete *Streptomyces lusitanus*

**DOI:** 10.3390/md10030668

**Published:** 2012-03-22

**Authors:** Zhuang Han, Ying Xu, Oliver McConnell, Lingli Liu, Yongxin Li, Shuhua Qi, Xiangzhong Huang, Peiyuan Qian

**Affiliations:** 1 Key Laboratory of Marine Bio-resources Sustainable Utilization, RNAM Center for Marine Microbiology, Guangdong Key Laboratory of Marine Material Medical, South China Sea Institute of Oceanology, Chinese Academy of Sciences, 164 West Xingang Road, Guangzhou 510301, China; Email: zhuanghan@ust.hk (Z.H.); shuhuaqi@scsio.ac.cn (S.Q.); 2 Division of Life Science, The Hong Kong University of Science and Technology, Clear Water Bay, Hong Kong, China; Email: boxuying@ust.hk (Y.X.); leonie@ust.hk (L.L.); liyongxing@ust.hk (Y.L.); xiangzhonghuang@yahoo.com.cn (X.H.); 3 Graduate School of the Chinese Academy of Sciences, Beijing 100049, China; 4 John I. Haas, Inc., Yakima, WA 98902, USA; Email: oliver.mcconnell@johnihaas.com

**Keywords:** marine-derived actinomycete, *Streptomyces lusitanus*, antibacterial, antimycin B

## Abstract

Two new antimycin A analogues, antimycin B1 and B2 (**1**–**2**), were isolated from a spent broth of a marine-derived bacterium, *Streptomyces lusitanus*. The structures of **1** and **2** were established on the basis of spectroscopic analyses and chemical methods. The isolated compounds were tested for their anti-bacterial potency. Compound **1** was found to be inactive against the bacteria *Bacillus subtilis*, *Staphyloccocus aureus*, and *Loktanella hongkongensis*. Compound **2** showed antibacterial activities against *S. aureus *and *L. hongkongensis* with MIC values of 32.0 and 8.0 μg/mL, respectively.

## 1. Introduction

Marine actinomycetes are chemically rich sources of structurally diverse secondary metabolites. The vast majority of these secondary metabolites is mainly derived from members of the genus *Streptomyces* [1,2]. Approximately 289 secondary metabolites from the marine-derived genus of *Streptomyces* are reported in the Marinlit database, covering a wide variety of chemical structures, including peptides, macrolides, lactones, indoles, terpenes, and quinones. These compounds show an extensive range of activities, such as cytotoxic, antibacterial, antifungal and antimalarial [3]. The bioactive secondary metabolites from marine-derived *Streptomyces* have thus attracted increasing interest during the last decades [4,5,6].

**Figure 1:**
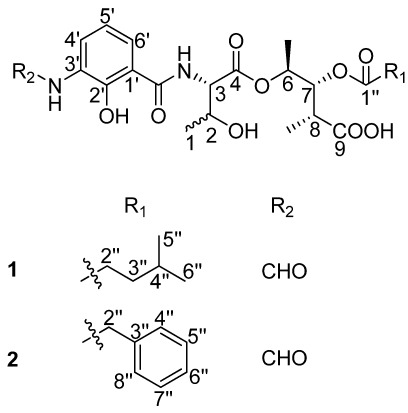
The structures of compounds **1** and **2**.

In our endeavor to search for novel antibacterial secondary metabolites from marine *Streptomyces* using bioassay and LC-UV/Vis-MS guided methods, we isolated two new antimycin A analogues. Antimycin A is characterized by the presence of a nine-membered dilactone ring, a carboxyl phenol amido unit and two alkyl side chains of varying lengths, with the only exception being antimycin A9, which has an aromatic 8-acyl residue [7]. The first group of antimycin A compounds was isolated as fungicides from *Streptomyces* sp. in 1949 [8]. Molecular studies showed that these compounds could inhibit the mitochondrial electron transport chain between cytochromes b and c [9]. To date, 20 antimycin A compounds have been reported [10]. A recent report analyzing the biosynthetic gene clusters of antimycin A from *Streptomyces* sp. S4 revealed that these compounds were synthesized by a hybrid NRPS/PKS [11]. Here, we report on the discovery of the first naturally occurring ring-opened antimycin A analogues which were named as antimycin B1 and B2 (Figure 1) as well as their antibacterial activity.

## 2. Results and Discussion

Compound **1** was obtained as a light-brown amorphous solid with a molecular formula of C_24_H_34_N_2_O_10_, which was deduced from NMR and HRESITOFMS data (Obsd [M + H]^+^ at *m/z* 511.2290), which suggested nine degrees of unsaturation. The UV spectrum indicated absorption at λ_max_ (ε) 220 (22,900) and 317 (5800) nm. The ^1^H NMR data for **1** (Table 1) indicated the presence of five exchangeable protons at δ_H_ 13.00 (s), 12.60 (s), 9.83 (brs), 8.81 (d, *J* = 8.0 Hz), and 5.09 (s), a formyl proton at δ_H_ 8.32 (d, *J* = 1.5 Hz), three aromatic protons at δ_H_ 8.27 (d, *J* = 8.0 Hz),7.80 (d, *J* = 8.0 Hz), and 6.92 (t, *J* = 8.0 Hz), four methine protons directly connected to heteroatoms δ_H_ 5.23 (dd, *J* = 10.0, 5.5 Hz), 4.98 (dq, *J* = 6.5, 5.5 Hz), 4.45 (dd, *J* = 8.0, 4.5 Hz), and 4.20 (m), one methine signal at δ_H_ 1.50 (m), and two methylene signals at δ_H_ 2.26 (m) and 1.37 (m) together with five methyl groups at δ_H_ 1.19 (d, *J* = 6.5 Hz), 1.16 (d, *J* = 6.5 Hz), 1.00 (d, *J* = 7.0 Hz), 0.84 (d, *J* = 7.0 Hz), and 0.82 (d, *J* = 6.5 Hz). These data suggested that **1** is an antimycin A analogue [8].

**Table 1:** ^1^H and ^13^C NMR data of compounds **1 **and **2 **(DMSO-*d*_6_, δ in ppm, *J* in Hz).

Position	Compound 1			Compound 2	
	δ_C_^a^	δ_H_ (*J* in Hz) ^b^		δ_C_^a^	δ_H_ (*J* in Hz) ^b^
1	20.04	1.16 d (6.5)		20.02	1.14 d (6.5)
2	65.7	4.20 m		65.7	4.16 m
2-OH		5.09 s			5.05 s
3	58.6	4.45 dd (8.0, 4.5)		58.7	4.45 dd (7.8, 4.3)
4	169.3			169.3	
6	69.3	4.98 dq (6.5, 5.5)		69.3	4.96 dq (6.5, 6.5)
7	73.0	5.23 dd (10.0, 5.5)		73.4	5.22 dd (7.0, 6.5)
8	38.8	2.69 m		38.8	2.67 m
9	174.5	12.60 s		174.6	12.60 s
6-Me	14.88	1.19 d (6.5)		14.59	1.14 d (6.5)
8-Me	11.22	1.00 d (7.0)		11.25	0.96 d (7.0)
1′	113.9			113.6	
2′	150.7			150.5	
3′	127.0			126.6	
4′	124.2	8.27 d (8.0)		124.3	8.26 d (8.0)
5′	117.8	6.92 t (8.0)		117.8	6.91 t (8.0)
6′	122.9	7.80 d (8.0)		122.1	7.77 d (8.0)
1′-CON*H*	170.0	8.81 d (8.0)		169.6	8.80 brs
2′-OH		13.00 s			13.00 s
3′-N*H*CHO		9.83 brs			9.83 brs
3′-NH*CH*O	160.5	8.32 d (1.5)		160.3	8.32 d (2.0)
1′′	172.2			170.5	
2′′	31.2	2.26 m		39.9	3.66 s
3′′	32.8	1.37 m		133.8	
4′′	26.6	1.50 m		129.1	7.23 d (8.0)
5′′	21.74	0.84 d (7.0)		128.0	7.31 t (8.0)
6′′	21.74	0.82 d (6.5)		126.6	7.25 t (8.0)
7′′				128.0	7.31 t (8.0)
8′′				129.1	7.23 d (8.0)

^a^ Recorded at 500 MHz; ^b^ Recorded at 125 MHz.

**Figure 2:**
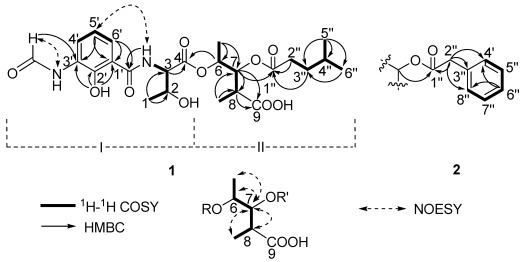
The key 2D correlations of compounds **1** and **2**.

Two substructures I and II were assigned by analyzing the 1D and 2D NMR spectra (Figure 2). Substructure I was assembled starting from the amino acid threonine moiety that was inferred from the ^1^H–^1^H COSY correlations of δ_H_ 4.20 (m, 2-H) with δ_H_ 4.45 (dd, *J* = 8.0, 4.5 Hz, 3-H) and δ_H_ 1.16 (d, *J* = 6.5, 1-H), of δ_H_ 4.45 (3-H) with δ_H_ 8.81 (d, *J* = 8.0 Hz, 1′-CON*H*) and HMBC correlations of 3-H with δ_C_ 169.3 (C-4). The ^1^H–^1^H COSY correlations from δ_H_ 6.92 (t, *J* = 8.0 Hz, 5-H′) to δ_H_ 8.27 (d, *J* = 8.0, 4-H′) and 7.80 (d, *J* = 8.0 Hz, 6-H′) indicated the presence of an 1,2,3-trisubstituted benzene. The HMBC correlations from phenolic hydroxyl proton observed at δ_H_ 13.00 (s, 2′-OH) to δ_C_ 113.9 (C-1′), 150.7 (C-2′) and 127.0 (C-3′), from the formamide proton observed at δ_H_ 9.83 (brs, 3′-N*H*CHO) to C-3′, from H-6′ to δ_C_ 170.0 1′-CONH, suggested that 2′-OH, 3′-NHCHO, and 1′-CONH groups were attached on C-2′, C-3′ and C-1′, respectively. HMBC correlations from H-6′, 3-NH and H-3 to the carbonyl carbon δ_C_ 170.0 (1′-CONH), allowed the trisubstituted aromatic ring moiety and the threonine moiety to be connected.

Substructure II was assembled from the following ^1^H–^1^H COSY and HMBC spectral data. ^1^H–^1^H COSY correlations from δ_H_ 4.98 (dq, *J* = 6.5, 5.5 Hz, 6-H) to δ_H_ 1.19 (d, *J* = 6.5 Hz, 6-Me) and δ_H_ 5.23 (dd, *J* = 10.0, 5.5 Hz, 7-H), from δ_H_ 2.69 (m, 8-H) to 7-H and δ_H_ 1.00 (d, *J* = 7.0, 8-Me) suggested the presence of a 2,3,4-trisubstituted pentanoic acid group. HMBC correlations from 7-H, 8-H and 8-Me to δ_C_ 174.5 (C-9) indicated a carbonyl group attached on C-8. ^1^H-^1^H COSY correlations from δ_H_ 1.50 (m, 4′′-H) to 0.84 (d, *J* = 7.0 Hz, 5′′-H), δ_H_ 0.82 (d, *J* = 6.5, 6′′-H) and δ_H_ 1.37 (m, 3′′-H), and from 3′′-H to δ_H_ 2.26 (m, 2′′-H), together with HMBC correlations from 3′′-H and 2′′-H to δ_C_ 172.2 (C-1′′) indicated the presence of a 4-methylvaleryl group. The long-range coupling between 7-H and C-1′′ revealed that the 4-methylvaleryl residue was connected to C-7 via an ester bond. The HMBC correlation of 6-H with C-4 allowed the substructures I and II to be connected.

There are five stereocenters in compound **1**. To assign the absolute configurations, two reactions were considered. The strategy was to establish the absolute stereochemistry of the threonine group using Marfey’s reagent, followed by lactonization to form the nine-membered dilactone ring found in cyclized analogs. If lactonization was successful, then NOEs could be obtained via NOESY or ROESY NMR experiments to complete the assignments. The threonine moiety of **1** was defined by acid hydrolysis and Marfey’s method [12,13], using standard amino acids, L-Threonine, L-*allo*-Threonine, D-Threonine and D-*allo*-Threonine. The derivatized threonine residue from compound **1** gave the same retention time as that prepared from standard L-Threonine and L-*allo*-Threonine (RT = 29.3 min). Thus, the absolute configuration of the C-3 was determined as 3*S*, but C-2 remains unresolved. The relative configurations at C-6, C-7 and C-8 were assigned as 6S*, 7R*, 8R* based on comparison of ^1^H and ^13^C chemical shifts and ^1^H-^1^H coupling constant data with literature values [14]. To determine the absolute configurations of C-2, C-6, C-7 and C-8, lactonization was attempted using a modification of Shiina’s method [15]. The mixture products were checked using LC-MS. However, no expected dehydrated molecule mass data was observed. Due to the limited amount of the compound, it was not possible to perform additional experiments to determine the absolute configurations of C-2, C-6, C-7 and C-8. Thus, the structure of **1** was determined as depicted in Figure 1; it is named antimycin B1.

Compound **2** had a molecular formula of C_26_H_31_N_2_O_10_ deduced from its HRESITOFMS data (Obsd [M + H]^+^ at *m/z* 531.1985) and NMR spectra. Comparison of the ^1^H and ^13^C NMR spectral data (Table 1) revealed close similarities between **2** and **1**. The difference between them was the absence of the 4-methylvaleryl residue in Substructure II of **1 **and the appearance of a phenyl-acetyl group in **2**, which is verified by HMBC correlations from δ_H_ 3.66 (s, 2′′-H) to carbonyl carbon δ_C_ 170.5 (C-1′′) and carbons on the second aromatic ring δ_C_ 133.8 (C-3′′), 129.1 (C-4′′, 8′′). The correlation between δ_H_ 5.22 (dd, *J* = 7.0, 6.5 Hz, 7-H) and C-1′′ revealed that the phenyl-acetyl residue was connected to C-7 by an ester bond. The absolute configuration of C-3 in **2** was also determined as 3*S* by applying Marfey’s method. The relative configurations at C-6, C-7 and C-8 were assigned as 6S*, 7R*, 8R* as in compound **1**. Thus, the structure of compound **2**, named antimycin B2, was determined and is also shown in Figure 1.

Compounds **1** and **2** were evaluated for their antibacterial activities against *Staphylococcus aureus*, *Bacillus subtilis* and *Loktanella hongkongensis*. Compound **1** did not show any activity against any of the three strains. Compound **2** showed selective activity against two strains, *S. aureus* and *L. hongkongensis*. See Table 2.

**Table 2:** Antibacterial activities of compounds **1** and **2**.

Compound	Antibacterial (MIC, μg/mL)		
	*Staphylococcus aureus*	*Loktanella hongkongensis*	*Bacillus subtillis*
**1**	NA	NA	NA
**2**	32.00	8.00	NA
Penicillin G	0.25	2.00	0.10
Streptomycin	8.00	16.00	8.00

NA means there is no bioactivity with a MIC > 32.00 μg/mL.

## 3. Experimental Section

### 3.1. General Experimental Procedures

The 1D and 2D NMR spectral data were obtained on Varian Inova 500 MHz NMR spectrometers. UV spectra were recorded on a Varian Cary 50 Conc UV Visible spectrophotometer with a path length of 1 cm. Optical rotations were measured using a Rudolph Research Autopol III Automatic polarimeter with a 10 cm cell. OD measurements of the antibacterial experiments were recorded at 600 nm on a Biorad Model 680 microplate reader. High-resolution mass spectra were acquired from UPLC-TOF-MS. The UPLC system was a Waters ACQUITY UPLC system (Waters, Manchester, UK) coupled to a Bruker microTOF-q II mass spectrometer (Brucker Daltonics GmbH, Bremen, German).

### 3.2. Isolation of Strain XM52, Identification, Cultivation, and Extraction

*Streptomyces* strain XM52 was isolated from the rhizosphere of the mangrove plant *Avicennia mariana* from Fujian Province, China. Strain XM52 was classified as a *Streptomyces lusitanus* based on nearly complete 16S rDNA analysis, which showed 99% sequence identity with *Streptomyces lusitanus* strain NBRC13464 (the sequence data has been deposited in GenBank under accession number NR 041143.1). The strain was cultured in 90 × 1.0 L volumes of GPY media (10.0 g glucose, 5.0 g peptone, 3.0 g yeast extract, 10.0 g sea salts, 1.0 L double distilled water, pH 7.2) while shaking at 160 rpm for 14 days at 23 °C. The fermented culture (90.0 L) was filtered through cheese cloth (8 layers) to separate the mycelia. The filtrate was extracted with ethyl acetate three times, while the mycelia were extracted by 80% acetone under ultrasonication. Evaporation of the acetone *in vacuo* left a wet residue that was partitioned with EtOAc. The combined two EtOAc extracts were evaporated *in vacuo* at 40 °C to yield 7.9 g of brown oily residue. The EtOAc extract (7.9 g) was subjected to reversed-phase C18 flash chromatography successively eluted with solvent mixtures of H_2_O-MeOH (9:1), H_2_O-MeOH (7:3), H_2_O-MeOH (5:5), H_2_O-MeOH (3:7), H_2_O-MeOH (1:9), and 100% MeOH. The H_2_O-MeOH (3:7) eluting fraction (labeled Fr. 4) showed potent antibacterial activity against *S. aureus*, *L. hongkongensis*, The Fr. 4 fraction was then subjected to Sephadex LH-20 eluted with MeOH to yield 15 sub-fractions (labeled Fr.4-1 to Fr.4-15), Fr. 4-12 were purified by C-18 semi-preparative HPLC (Phenomenex Luna C18 (2) 250 × 10 mm column) with MeCN-H_2_O (38:62–42:58) to obtain pure compounds **1** (3.5 mg) and **2** (2.4 mg).

Antimycin B1 (**1**): Light-brown amorphous solid, 3.5 mg, [α]^25^_D_ = 0 (*c* 0.1, CH_3_OH); UV (MeCN) λ_max_ (ε): 220 (22,900) and 317 (5800) nm. HRESIMS: Obsd *m/z* 511.2290 [M + H]^+^ (C_24_H_35_N_2_O_10_ requires 511.2292). Table 1 presents the ^1^H and ^13^C NMR data.

Antimycin B2 (**2**): Light-brown amorphous solid, 2.4 mg, [α]^25^_D_ = 0 (*c* 0.1, CH_3_OH); UV (MeCN) λ_max_ (ε): 220 (23,300) and 317 (5900) nm. HRESIMS: Obsd *m/z* 531.1985 [M + H]^+^ (C_26_H_31_N_2_O_10_ requires 531.1979). Table 1 presents the ^1^H and ^13^C NMR data.

### 3.3. Marfey’s Analysis of the Threonine Moiety

A 0.2 mg-portion of compound **1** was placed in a sealed glass tube, dissolved in 6 N HCl (1.0 mL) and heated to 110 °C for 20 h. Hydrolysates were evaporated to dryness and then resuspended in water (40.0 μL). A solution of (1-fluoro-2,4-dinitrophenyl)-5-L-alanine amide (FDAA) (4.2 μmol) in acetone (150.0 μL) and then 1 N NaHCO_3_ (20.0 μL) was added to each reaction vessel and the mixtures were stirred at 40 °C for 2 h. A 2 N HCl solution (10.0 μL) was added to each reaction vessel to stop the reaction and the solution was evaporated *in vacuo*. The residues were then resuspended in 200.0 μL of MeOH and subjected to HPLC (Phenomenex Luna C18 (2) 250 × 4.5 mm column) using 50 mM TEAP/acetonitrile linear gradient elution (pH 3.0, from 10% to 40% acetonitrile during 45 min, flow rate 1.0 mL/min, at 340 nm), four standard Threonine-FDLA derivatives that had been prepared using the same method were compared. HPLC analysis of Marfey’s derivatives from the direct hydrolysis of **1** established the following retention times of the derivatized amino acids (reference derivatives *t*_R_): L-Thr 29.3 min (L-Thr 29.3 min, L-*allo*-Thr 29.3 min, D-Thr 31.6 min, D-*allo*-Thr 31.6 min).

### 3.4. Lactonization

A solution of compound **1** (2.0 mg) in dry toluene (1.0 mL) was slowly added over 7 h through a syringe pump to a toluene solution (0.5 mL) of 2-methyl-6-nitrobenzoic anhydride (MNBA) (1.3 mg, 3.75 μmol), 4-(dimethylamino) pyridine (DMAP) (1.4 mg, 15.0 μmol), stirred at room temperature under an atmosphere of N_2_. After the completion of the addition, the resulting mixture was stirred for another 13 h. The reaction mixture was then centrifuged. The suspension was diluted with EtOAc(50 mL), washed with aqueous saturated NaHCO_3_, water, and brine, and dried over anhydrous Na_2_SO_4_. The dried sample was then subjected to LC-MS to check the products.

### 3.5. Evaluation of Antibacterial Activity

The antibacterial activities of compounds **1** and **2** were evaluated by MIC assays against *Staphylococcus aureus*, *Bacillus subtilis*, and *Loktanella hongkongensis*. Briefly, the bacterial strains were inoculated in YP Broth (0.2% yeast extract, 0.1% peptone, 1.7% sea salts) and were incubated at 28 °C for 12 h. A stock solution of the sample was prepared at 50 mg/mL in DMSO and further diluted to varying concentrations in 96 well plates that contained the incubated microbial strains. The plates were incubated at 28 °C overnight. Cell growth was checked by measuring the optical density at 600 nm; growth inhibition was compared to that caused by varying concentrations of Penicillin G and Streptomycin (serving as the positive controls).

## 4. Conclusions

Two new antimycin A analogues, antimycin B1 (**1**) and antimycin B2 (**2**), were isolated from a spent broth of marine-derived actinomycete *Streptomyces lusitanus*. The key structural features of compounds **1** and **2** were characterized by spectroscopic analyses and chemical methods. The compounds were suspected to be artificial products originated during the treatment process. A previous degradation study of antimycin A compounds revealed that mild alkaline hydrolysis of antimycin A compounds yielded blastmycic acid and antimycin lactone, while blastmycic acid would be further degraded into antimycic acid under more severe conditions [16]. However, due to intramolecular reaction [17], no ring-opened dilactone products were observed. Thus, the antimycin B compounds described here are the first naturally occurring antimycin A dilactone opened products to be identified. Antibacterial activities were evaluated using MIC assays. Compound **2** showed moderate antibacterial activities against *S. aureus*, *L. hongkongensis* and *B. subtilis*, with the activity against *L. hongkongensis* being stronger than that of streptomycin. Compound **1** did not show any activities against these three bacterial strains.
